# Multi-wave analyses of coping, athlete burnout, and well-being among F. A. Premier League academy players

**DOI:** 10.3389/fpsyg.2022.979486

**Published:** 2022-08-11

**Authors:** Adam R. Nicholls, Daniel J. Madigan, Keith Earle

**Affiliations:** ^1^Department of Sport, Health, and Exercise Science, University of Hull, Hull, United Kingdom; ^2^School of Science, Technology, and Health, York St. John University, York, United Kingdom

**Keywords:** exhaustion, mental health, stress, task-oriented, coping

## Abstract

Being a player with an F. A. Premier football academy is very prestigious for young players, but it can also be very stressful too. Coping with stress is particularly important given that one of the undesirable consequences linked to chronic stress is athlete burnout, which may also negatively impact psychological well-being. Understanding the most effective ways to cope with stress, therefore, is important for optimizing academy athlete education. Consequently, the aim of the present study was to examine whether coping predicted changes in athlete burnout, and whether athlete burnout predicted changes in well-being across 14 weeks of the competitive season. A sample of 26, under-18 and under-23, male F.A. Premier academy athletes completed weekly assessments of coping (task-, distraction-, and disengagement-oriented), athlete burnout, and psychological well-being on 14 separate occasions. The results of within-person analyses revealed that task-oriented coping predicted decreases in athlete burnout, which in turn predicted decreased well-being. Teaching high-level academy athletes task-oriented coping strategies may be useful in reducing athlete burnout, which may additionally protect athletes' well-being.

## Introduction

All teams that compete in the F. A. Premier League, except for Brentford Football Club, have a football academy. The aim of these academies is to increase the number of players who play in the first teams of Premier League clubs. They do so by offering world class coaching that produces tactically and technically excellent players, effective decision makers, and educationally rounded players (Football Association Premier League, 2011). Although representing an F.A. Premier League academy is an excellent opportunity, it can be very stressful for young athletes (Reeves et al., 2011a). Constructs linked to stress among young athletes include burnout, coping, and well-being (Hill et al., 2010; Madigan et al., 2019, 2020). To date, however, researchers have used cross-sectional data collection (Hill et al., 2010) or in-frequent measurements of coping and athlete burnout (e.g., Madigan et al., 2020), which may not provide an accurate insight into the relation between these two constructs. Indeed, Lazarus (1999) argued that when assessing the relationship between coping and other constructs, proximity and the frequency of assessment is important to establish more valid findings. Additionally, although the well-being and stress relationship is established, little is known about its association with athlete burnout. The aim of the present study was to examine the extent to which coping predicted changes in athlete burnout and whether changes in athlete burnout are linked to decreased well-being, measured on a weekly basis, across 14 weeks of the most stressful part of the season (Reeves et al., 2011b).

### Stress in sport

Players within football academies have reported a range of performance and non-performance stressors. Performance stressors relate to making a mistake during a match, team performance, selection, and individual performance. Non-performance stressors include contractual issues, being evaluated by academy managers and coaches, and future opportunities to play in the Premier League (Reeves et al., 2009). Securing a professional contract can be stressful, because the number of players vying for a contract exceeds the number of contracts available (Reeves et al., 2011b). As such, many players will be de-selected and not offered a professional contract. Experiencing chronic stressors, such as those encountered by the players in Reeves et al. (2011b), can lead to burnout (Lin et al., 2021), which is linked to poorer well-being (Madigan et al., 2019). Given the importance of coping for both sporting performance (e.g., Nicholls et al., 2016a) and well-being (e.g., Nicholls et al., 2016b), understanding more about the relationship between coping and athlete burnout, along with athlete burnout and well-being is warranted.

### Coping in sport

A construct that can help athletes manage stress is coping. Coping refers to all cognitive and behavioral attempts to manage internal or external demands that have been appraised as stressful (Lazarus, 1999). It can be measured as a process to reveal how an athlete coped in a specific situation or situations, or it can be measured dispositionally to indicate how a person normally copes, and thus coping tendencies (Lazarus, 1999). Both approaches to assessing coping having their strengths and weaknesses. That is, dispositional approaches fail to capture changes in momentary behavior, but are excellent at predicting trends in coping. Process approaches to assessing coping accurately capture how an athlete copes in a situation, but fails to predict coping over long stretches of time (Fleeson, 2004). As such, both approaches to assessing coping are acceptable, depending on the research question.

Coping is linked to a variety of desirable outcomes among athletes, such as increased resilience (Thompson et al., 2021), sporting performance (Nicholls et al., 2016a), well-being (Nicholls et al., 2016b), but also undesirable consequences such as symptoms of depression and burnout (Nixdorf et al., 2013; Madigan et al., 2020). The most widely used conceptualization of coping in sport, according to Nicholls et al. (2016a), is a three-factor approach that was proposed by Connor-Smith et al. (2000) and Walker et al. (1997). This was adapted for sport by Gaudreau and Blondin (2004), who proposed task-, distraction-, and disengagement-oriented coping. Task-oriented refers to coping attempts to master a stressful situation. Distraction-oriented coping is when athletes focus on cues that are not sport relevant. Disengagement-oriented coping is when an athlete stops striving for his or her personal goals. Although two-factor coping classifications are also widely used among athletes (Nicholls and Polman, 2007), such as problem- (i.e., active efforts to manage situations) and emotion-focused coping (i.e., efforts to regulate emotional responses; Lazarus, 1999), three factor-classifications capture the structure of coping much more effectively (Connor-Smith et al., 2000; Compas et al., 2001).

### Athlete burnout

According to Raedeke and Smith (2001), athlete burnout comprises of three specific symptoms: (a) a reduced sense of accomplishment in which an athlete negatively evaluates his or her previous achievements, (b) an athlete devaluing or resenting his or her sport, and (c) athletes experiencing physical and emotional exhaustion. Athlete burnout is extremely problematic because it associated with many undesirable consequences such as an increased risk of depression, poorer personal relationships, reduced performance, and dropout (Larson et al., 2019; Smith et al., 2019). Worryingly, a cross-temporal meta-analysis revealed that average levels of a reduced sense of athletic accomplishment and sport devaluation have linearly increased over the last two decades (Madigan et al., 2022).

Understanding athlete burnout is crucial for reducing its prevalence. To this end, scholars have developed models to explain this process. The most widely cited study on burnout, according to Madigan et al. (2020), is (Smith, 1986) cognitive-affective model. In this model, chronic stress is the antecedent of burnout, via appraisals of coping. When athletes appraise that their ability to deal with the stress is outweighed by the demands of the situation, stress will ensue (Lazarus, 1999). As such, the imbalance between coping resources and the demands of sport can cause burnout. Support for Smith's model was recently provided by Lin et al. (2021) who conducted a systematic review and meta-analysis of 44 studies and confirmed the stress and burnout relationship.

### Coping and athlete burnout

From a theoretical perspective, coping and athlete burnout are related constructs, given that stress is associated with burnout, and coping is a mechanism of reducing stress (Raedeke and Smith, 2001, 2004). Coping is, however, differentially associated with burnout. In two cross-sectional studies by Hill et al. (2010) and Pacewicz et al. (2018), and a two-wave longitudinal study by Schellenberg et al. (2013), avoidance coping was positively associated with burnout. Problem-focused coping was significantly and negatively associated within burnout in two of the studies (e.g., Hill et al., 2010; Schellenberg et al., 2013), but not the Pacewicz et al. study. In another study, Madigan et al. (2020) examined coping and burnout at the start, middle, and end of the season among academy athletes. Although Madigan et al. also reported that avoidance coping positively predicted athlete burnout, problem-focused coping was unrelated to changes in athlete burnout.

At the present time, there is a theoretical link between coping and burnout (e.g., Raedeke and Smith, 2001, 2004), but the empirical findings for specific coping strategies (e.g., problem-focused) are equivocal. This may be due to an over reliance on cross-sectional designs (Hill et al., 2010), the structure of coping not being accurately assessed in coping and athlete burnout studies (e.g., Compas et al., 2001), or relatively few assessments of coping and burnouts in longitudinal studies (e.g., Schellenberg et al., 2013; Madigan et al., 2020; Pires and Ugrinowitsch, 2021a,b ). More frequent assessments among athletes and assessing coping as a three-factor structure may therefore provide a more accurate account of the relationship between athlete burnout and coping.

### Athlete burnout and well-being

Well-being is the extent to which people fulfill their abilities, cope with stressors in life, and work productively or fruitfully (World Health Organisation, 2004), and includes both a hedonic and eudaimonic component. The Hedonic perspective relates to happiness and pleasure attainment, whereas the eudaimonic perspective relates to the degree to which a person can function fully (Ryan and Deci, 2001; Tennant et al., 2007). Although Madigan et al. (2019) theorized that burnout affected well-being, thus far, researchers have failed to establish links between well-being and athlete burnout. An intervention study by Dubuc-Charbonneau and Durand-Bush (2015) found that a self-regulatory intervention increased well-being and reduced athlete burnout across four phases of a season, but it did not report the link between the two constructs. In another study, Thomas et al. (2021) examined the impact of the talent development environment on well-being and athlete burnout but failed to report whether athlete burnout and well-being were related. Despite Eklund et al. (2011) advocating the importance of understanding the relationship between variables such as well-being and athlete burnout, little is known about this relationship. To fully understand athlete burnout, it is important that researchers explore how it is related to optimal functioning such as well-being. Although the relationship between well-being and athlete burnout has not been examined, Veronese et al. (2022) found that well-being and burnout were negatively associated among a sample of health-care providers.

### The present study

The aim of the present study was to examine whether weekly assessments of coping predicted changes in athlete burnout, and then whether changes in burnout predicted well-being among a sample of F. A. Premier League academy football players. Due to the equivocal nature of the relationship between coping and athlete burnout, the over reliance on cross-sectional data, and scholars using Lazarus' (1999) classification of coping, forming hypotheses was not straightforward. We formulated our hypotheses based on previous studies (e.g., Hill et al., 2010; Schellenberg et al., 2013; Madigan et al., 2020), and predicted that task-oriented coping would negatively predict changes in athlete burnout, whereas distraction- and disengagement-oriented coping would positively predict athlete burnout. Based on Madigan et al., 2019 assertion that athlete burnout influences well-being, we predicted that burnout would negatively predict well-being.

## Method

### Participants

Twenty six male players from an F.A. Premier League academy participated in the present study. Players represented the under-18 (*n* = 15) or the under-23 (*n* = 11) team. The players were aged between 17 and 21 years (*M* age = 18.4; *SD* = 1.20)^1^.

### Procedure

This study received ethical approval from a university ethics committee, and informed consent was obtained for all participants. Players within the academy received a study information letter and a consent form, which was distributed by the third author. Parental consent was obtained for all participants aged below 18 years. Participants were administered measures of coping, athlete burnout, and well-being at the club's training ground for 14 weeks, in the presence of the third author. Data was collected during the months of September, October, November, and December, given previous research by Reeves et al. (2011a) indicating that these are the most stressful periods for F.A. Premier League academy players.

### Measures

#### Coping

Coping was assessed using a single item for the three second-order dimensions of the Coping Inventory for Competitive Sport (CICS; Gaudreau and Blondin, 2002). We assessed three second-order dimensions, in accordance with Doron and Gaudreau (2014). The stem for task-oriented coping was “Task-oriented coping represents the means that you are using to manage a game situation or to solve a problem you are facing in the match or training. It includes efforts to concentrate, to seek information or advice from the training staff, to analyse the point, to manage your time in a point, to enhance your effort, to manage your goals, to identify solutions, to create and use a plan of actions to make your actions more efficient.” Players responded to the following stem for distraction-oriented coping “Distraction-oriented coping corresponds to the strategies that can be used to direct one's attention momentarily on things that are unrelated to sport competition, such as keeping your distance from other players or coaches, or thinking about other things to distract yourself, such as friends and family.” The disengagement-oriented stem was “Disengagement-oriented coping represents strategies that are used to disengage oneself from the process that could generally leads to goal attainment such as stopping believing you could achieve your goal, wishing competition or training would end, or getting angry.” Players were asked to rate the extent to which task-, distraction-, and disengagement-oriented coping corresponded to what they did to cope in training or matches by circling the appropriate number. These questions were answered on a 5-point Likert-type scale anchored at 1 (*not at all*) and 5 (*very strongly*).

#### Athlete burnout

The 15-item Athlete Burnout Questionnaire (ABQ; Raedeke and Smith, 2001) assessed athlete burnout among the sample. The ABQ comprised of three subscales, which each have five questions, and captured a reduced sense of accomplishment (e.g., “I am not achieving much in my sport”), physical and emotional exhaustion (e.g., “I am exhausted by the mental and physical demands of my sport”), and devaluation (e.g., “I'm not into my sport like I used to be”). The subscales were combined to create a total score of athlete burnout. Participants were asked how often they experienced the symptoms described in the statements responding on a scale from 1 (*almost never*) to 5 (*almost always*) throughout the previous week. Evidence for the reliability and validity of the ABQ has been provided by Raedeke and Smith (2001).

#### Well-being

The 7-item Short Warwick Edinburgh Mental Well-Being Scale (SWEMWBS; Tennant et al., 2007) examined well-being among the participants. Participants responded to the stem “Below are some statements about feelings and thoughts. Please circle the number that best describes your experience of each over the last week.” The SWEMWBS includes both the hedonic and eudaimonic perspective of well-being and includes questions such as (i.e., “I've been feeling relaxed” and “I've been dealing with problems well”), which are answered on a 5-point Likert-type scale ranging from 1 (*none of the time)* to 5 (*all of the time*). Evidence for the reliability and validity of the SWEMWBS has been provided by Tennant et al. (2007).

### Analytical strategy

**Figure 1:**
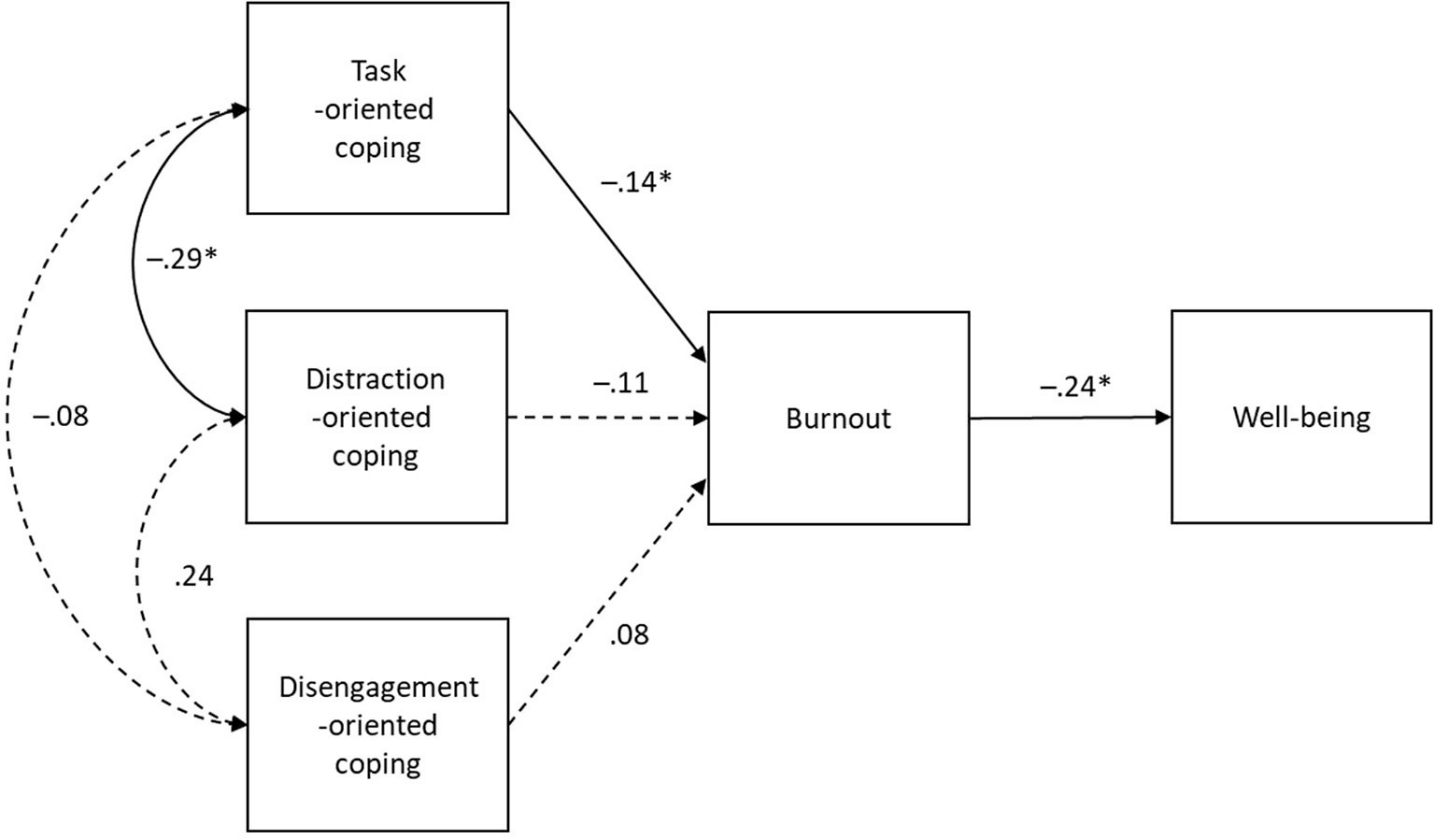
Within-person model illustrating relationships between changes in coping strategies, athlete burnout, and well-being. *p < 0.05. Dashed lines represent non-significant paths (*p* > 0.05).

To examine whether coping predicted changes in athlete burnout, and whether burnout was related to changes in well-being, we used multilevel path analysis with the measurement occasions (T1–T14) representing the within-person level. We focused our analyses on the within-person level (changes) and so multilevel path analysis provided the means to disaggregate the levels of analyses (see Laporte et al., 2021). Robust Maximum Likelihood in Mplus 7.0 was used (Muthén and Muthén, 1998–2012) to test the models accompanied by the mean-adjusted chi-squared test statistic. Because our interest was in the within-person part of the model, our model fit evaluation included markers of overall model fit (i.e., RMSEA), but focused on markers of fit for the within-person aspect (i.e., SRMRwithin). Acceptable fit was based on the following benchmarks root mean square error of approximation (RMSEA) < 0.10, and the standardized root mean square residual (SRMR) < 0.10 (e.g., Byrne, 2013). More importantly, we were interested in the size and significance of paths in the within-person model (see Figure 1).

## Results

### Preliminary analyses

Data were screened following the protocol outlined by Tabachnick and Fidell (2014) using IBM Statistics SPSS 25.0. Across the 14 time points, missing value analysis indicated that there were 276 complete cases and 88 missing responses. In these instances, we used the full information maximum likelihood (FIML) method for model estimation for missing data (Enders and Bandalos, 2001). Next, subscales were then computed and screened for univariate (Z scores) and multivariate outliers (Mahalanobis distance). These assessments did not result in any further cases being removed from the study.

### Multilevel path analysis

#### Intraclass correlations

To determine the amount of variance attributable to the different levels (within vs. between), we calculated the intraclass correlations, which were: Task-oriented coping = 0.52, distraction coping = 0.47, disengagement coping = 0.63, athlete burnout = 0.67, and well-being = 0.62. As a rule, data are suitable for multilevel path analysis when intraclass correlation coefficients are above 0.05 (Preacher et al., 2010).

#### Within person model

The within-person model provided acceptable model fit (χ2 [13] = 47.59, scaling factor = 1.02, SRMRwithin = 0.05, RMSEA = 0.098). The model showed task-oriented coping predicted decreases in distraction coping, but was unrelated to disengagement coping. Distraction- and disengagement-oriented coping were unrelated. Task-oriented coping predicted decreases in athlete burnout, but distraction and disengagement were unrelated to changes in burnout. Finally, athlete burnout predicted decreased well-being. See Figure 1 for the full model.

## Discussion

The aim of the present study was to examine whether coping predicted changes in athlete burnout, and whether changes in athlete burnout predicted well-being among a sample of F. A. Premier League academy players. We found partial support for hypotheses, because task-oriented coping negatively predicted athlete burnout, and athlete burnout negatively predicted well-being. Distraction- and disengagement-orient coping, however, were not associated with athlete burnout.

### Coping and athlete burnout

The literature regarding the relationship between coping and athlete burnout is complex. For example, problem-focused coping has been both negatively and unrelated to athlete burnout, and avoidance coping has been negatively associated with burnout (e.g., Hill et al., 2010; Schellenberg et al., 2013; Madigan et al., 2020). In the present study, unlike previous studies that examined the coping and athlete burnout relationship, we assessed coping using the three-factor approach. As such, making direct comparisons between the present study and previous research is not straightforward. Our findings do however agree with some previous cross-sectional studies who found a negative relationship between problem-focused coping and athlete burnout (e.g., Hill et al., 2010; Schellenberg et al., 2013).

It is worthwhile considering why task-oriented coping was associated with fewer athlete burnout symptoms among the players in the present study. One of the dimensions of burnout is a reduced sense of accomplishment and therefore the extent to which athletes perceive their level of achievement in sport. A meta-analysis that examined the relationship between coping and sporting performance revealed that mastery coping strategies, which captures both task-oriented and problem-focused coping, were associated with higher levels of sporting performance (Nicholls et al., 2016a). As such, it is highly plausible that the task-oriented coping strategies helped the athletes to maintain their performance levels and thus minimized symptoms of athlete burnout.

Another possible explanation regarding beneficial effects of task-oriented coping on athlete burnout symptoms relates to the emotional exhaustion dimension of burnout. One of the coping strategies in the task-oriented coping dimension is relaxation and includes breathing and physical relaxation strategies to increase how relaxed an athlete feels. With a sample of health healthcare professionals, Schmid and Thomas (2021) found that relaxation was negatively linked to emotional exhaustion. Future work should therefore explore this link and expand it to include other plausible psychophysiological mechanisms.

### Athlete burnout and well-being

In support of theoretical predictions (i.e., Madigan et al., 2020) and research in non-sport settings (i.e., Veronese et al., 2022), we found that athlete burnout was negatively associated with well-being. Conceptually, this makes sense because athlete-burnout is linked to stress (Lin et al., 2021), so when athletes are experiencing burnout symptoms, they have likely experienced high levels of stress over a prolonged period, and stress is itself associated with lower well-being (Nicholls et al., 2016b). Given these findings, more emphasis should be placed on reducing burnout in athletes. This should be a high priority because lower well-being is associated with undesirable consequences such as social disconnectedness, anxiety, depression, and damage to self-esteem (Bartholomew et al., 2011; Marsters and Tiatia-Seath, 2019).

### Applied recommendations

To reduce symptoms of burnout among young male athletes within professional academies, sports psychologists and coaches could provide training on task-oriented coping strategies. Task-oriented coping includes logically analyzing stressful situations to identify how master the situation, thought control strategies, how seek support from other people, relaxation strategies, and mental imagery. Previous studies in this area have shown that such interventions are effective in regard to increasing the effectiveness of coping strategies to reduce stress (see e.g., Reeves et al., 2011a), but further research regarding athlete burnout is necessary to confirm if this as a viable approach. Coaches and psychologists could also monitor well-being throughout the season, given that stressor frequency appears to fluctuate among young athletes in F.A. Premier League Academies, with early- and mid-season being the phases of the season in which the most stressors are reported (Reeves et al., 2011b).

### Limitations and future research

The present study has several limitations. For example, it is unclear whether findings of the current research could be generalisable to females athletes, individual sport athletes, or those not involved with a professional academy. Further research is required to address this issue, so that recommendations can be provided for females and athletes at other levels. Another possible limitation of this study is the sample size, which is smaller than the other studies that assessed coping and athlete burnout (e.g., Hill et al., 2010; Schellenberg et al., 2013). Future work may wish to take advantage of recent advances in generalizability theory (e.g., Blanco-Villaasenor et al., 2014) and design studies to isolate and estimate as many facets of measurement error as possible (see also Webb and Shavelson, 2005). It should be noted that although there were only 26 participants in this study, the data was collected weekly over a period of 14 weeks and resulted in 276 data captures. Smaller sample sizes are generally observed in studies with elite athletes or when intensive longitudinal designs are employed. Future work is required to confirm the findings with larger samples, but to do so will require the collaborative efforts of large groups of researchers and the support of sports clubs and organizations.

## Conclusion

In the present study, we found partial support for our model, as task-oriented coping predicted decreases in athlete burnout. Consequently, teaching athletes how to use task-oriented coping strategies may be effective at reducing symptoms of burnout across extended periods of time. Finally, we found that athlete burnout was associated with reduced well-being, which reiterates the detrimental impact burnout can have for athletes.

## Data availability statement

The raw data supporting the conclusions of this article will be made available by the authors, without undue reservation.

## Ethics statement

The studies involving human participants were reviewed and approved by Faculty of Health Science, University of Hull. Written informed consent to participate in this study was provided by the participants' legal guardian/next of kin.

## Author contributions

AN and KE conceptualized the study. DM conducted the statistical analyses. All authors contributed to writing the manuscript. All authors contributed to the article and approved the submitted version.

## Conflict of interest

The authors declare that the research was conducted in the absence of any commercial or financial relationships that could be construed as a potential conflict of interest.

## Publisher's note

All claims expressed in this article are solely those of the authors and do not necessarily represent those of their affiliated organizations, or those of the publisher, the editors and the reviewers. Any product that may be evaluated in this article, or claim that may be made by its manufacturer, is not guaranteed or endorsed by the publisher.

## Footnotes

^1^The sample size was inline with previous studies using multilevel analyses (e.g., Li et al., 2018) and examining longitudinal changes in burnout (e.g., Pires and Ugrinowitsch, 2021a).
